# Surface chemistry for cytosolic gene delivery and photothermal transgene expression by gold nanorods

**DOI:** 10.1038/s41598-017-04912-1

**Published:** 2017-07-05

**Authors:** Hirotaka Nakatsuji, Kelly Kawabata Galbraith, Junko Kurisu, Hiroshi Imahori, Tatsuya Murakami, Mineko Kengaku

**Affiliations:** 1 Department of Molecular Engineering, Graduate School of Engineering, Kyoto University, Nishikyo-ku, Kyoto 615-8510 Japan; 2 Graduate School of Biostudies, Kyoto University, Sakyo-ku, Kyoto 606-8501 Japan; 3 Institute for Integrated Cell-Material Sciences (WPI-iCeMS), Kyoto University, Sakyo-ku, Kyoto 606-8501 Japan; 4 Department of Biotechnology, Faculty of Engineering, Toyama Prefectural University, Kurokawa 5180, Imizu City, Toyama 939-0398 Japan

## Abstract

Light-inducible gene regulation has great potential for remote and noninvasive control of the fate and function of target cells. One method to achieve such control is delivery of heat shock protein (HSP) promoter-driven protein expression vectors and photothermal heaters into the cells, followed by activation by illumination. In this study, we show that gold nanorods (AuNRs) functionalized with two conventional lipids, oleate and 1,2-dioleoyl-3-trimethylammonium-propane (DOTAP), are capable of efficient transfection and quick photoactivation of the HSP promoter. Use of our AuNRs (DOTAP-AuNRs) was comparable to Lipofectamine 2000 in terms of transfection efficiency, while lower in cytotoxicity. Subsequent near-infrared laser (NIR) illumination of the cells transfected by DOTAP-AuNRs for 10 s induced time- and site-specific transgene expression without significant phototoxicity, to a degree similar to that of heating the entire culture dish for 30 min. Our mechanistic studies suggest that efficient transfection and quick photoactivation of the HSP promoter (HSP70b’) are due to the promoted endosomal escape of DOTAP-AuNRs. We propose a novel protocol for NIR-inducible, site-directed gene expression using an unprecedented complex of the three conventional components capable of both transfection and photothermal heating.

## Introduction

Optical control of gene expression is an attractive strategy to study the function of genes of interest as well as to develop novel cancer therapy^1–4^. One of the modalities to achieve such control is use of the photothermal effect as a trigger to activate heat shock protein (HSP) promoters^5–12^. In vertebrates and invertebrates, HSP promoters are regulated by heat shock factor (HSF), a transcription factor that localizes in the cytosol in a dormant state under unstressed condition. HSF is activated in response to various cellular stresses, including heat^13^, and induces the expression of downstream HSPs, whose functions are important for cellular defense. Therefore, activation of HSP promoter-driven gene expression by laser light is a powerful approach for spatial and temporal control of protein expression. In addition, use of a laser wavelength in the near-infrared (NIR) region (650–900 nm) for therapy may reduce the invasiveness of the photoactivation of HSP promoters, due to its reduced absorption by water and hemoglobin^14^.

NIR activation of HSP promoters in mammalian cells have been previously reported in studies using NIR-responsive nanomaterials, such as carbon nanohorns^10^, silica-gold nanoshells^11^, hollow gold nanoparticles^11^, and gold nanorods (AuNRs)^12^. In these studies, HSP promoter-driven vectors and NIR-responsive nanomaterials were separately introduced into cells, prior to NIR laser illumination for the nanomaterial-mediated photothermal heating of the cells. Among the above-mentioned nanomaterials, AuNRs have also been developed as gene delivery carriers via surface modification with cationic polymers^15–18^. However, its relative transfection efficiency and feasibility as an intracellular photothermal heater have never been presented. A possible reason for the latter may be the lack of knowledge regarding the surface chemistry of AuNRs capable of both efficient transfection and safe photoactivation. Given that the threshold temperature for HSP promoter activation and cell death is almost the same (ca. 42 °C)^19, 20^, a highly sensitive intracellular photothermal heating system is required for safe activation of HSP promoters. In this context, we previously reported that the control of the surface chemistry of AuNRs was essential to achieve safe photothermal heating of the plasma membrane over 43 °C^21^. In the present study, we prepared a series of surface-modified AuNRs with different cationic dispersants and found that AuNRs functionalized with 1,2-dioleoyl-3-trimethylammonium-propane (DOTAP) showed comparable transfection efficiency to a commonly used transfection reagent, Lipofectamine 2000. Moreover, DOTAP-treated AuNRs (DOTAP-AuNRs) can efficiently photoactivate the concurrently transfected HSP promoter-driven vector with brief NIR illumination (10 s), without significant cytotoxicity.

## Results

### Preparation of AuNR/plasmid DNA complexes

**Figure 1:**
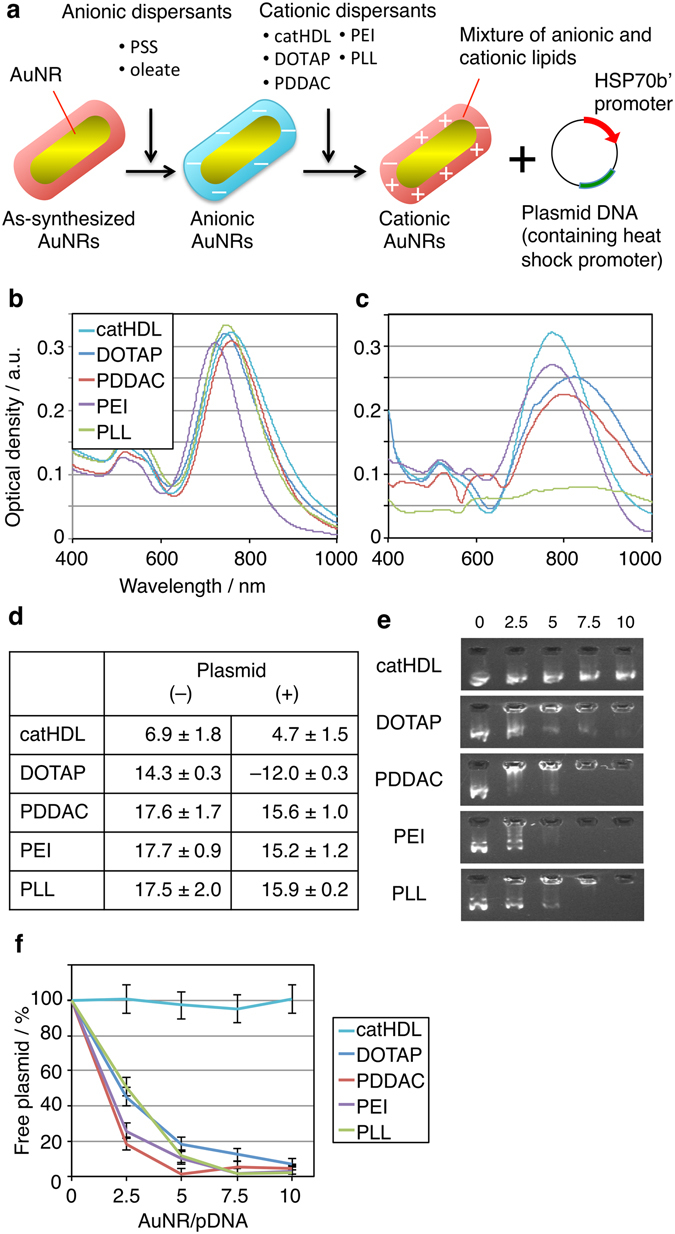
Characterization of cationic AuNRs. (**a**) Schematic of preparation of cationic AuNR/pDNA complex. (**b**) UV-*vis*-NIR absorption spectra of various cationic AuNRs in PBS ([Au] = 20 µg/mL). All AuNRs have intense absorption in the NIR region, suggesting their high colloidal stability. (**c**) UV-*vis-*NIR absorption spectra of various cationic AuNRs after complexation with control plasmid DNA (pCMV-DsRed) in Opti-MEM ([Au] = 20 µg/mL). AuNR/pCMV-DsRed complexes were prepared by simply mixing AuNRs and pCMV-DsRed (w/w ratio = 10) for 20 min at room temperature. Significant broadening of the NIR plasmon peak is observed only for PLL-AuNRs. (**d**) Zeta potential data (mV, n = 3, average ± SD) of AuNRs before and after complexation with pCMV-DsRed in 10 mM Tris-HCl (pH 7.4). Only DOTAP-AuNRs show a large decrease in the zeta potential upon pCMV-DsRed addition. (**e**) Agarose gel shift assay. AuNRs were added to 10 µg/mL pCMV-DsRed solution at various w/w ratios. pCMV-DsRed was visualized by Gel Green. Numbers in the image indicate the w/w ratios of AuNRs to pCMV-DsRed. (**f**) Densitometric analysis of gel bands in (**e**). Values were calculated from the residual fluorescence intensity of free pCMV-DsRed (n = 3, average ± SD).

As-synthesized AuNRs^22^ were first treated with oleate^23^ or poly(styrene sulfonate) (PSS)^17^, followed by one of the following cationic dispersants: DOTAP, poly(diallyldimethylammonium chloride) (PDDAC), polyethyleneimine (PEI), poly-L-lysine (PLL), or cationized HDL (catHDL) (Fig. 1a). We have previously reported that catHDL is a highly biocompatible and cell-interactive dispersant for AuNRs^23^. Oleate-treated AuNRs (oleate-AuNRs)s were only used for catHDL and DOTAP, because the other cationic polyelectrolytes caused aggregation (Supplementary Fig. S1). DOTAP-AuNRs were prepared first by mixing oleate-AuNRs and DOTAP/sodium cholate micelles at a DOTAP/AuNR weight ratio of 10, followed by dialysis against PBS to remove cholate. All cationic AuNRs had intense absorption in the near infrared region, suggesting good colloidal stability in PBS (Fig. 1b). The presence of the cationic dispersant on the AuNR surface was confirmed by Zeta potential analysis (Fig. 1d) and IR spectroscopy (Supplementary Fig. S2).

Next, the pDNA-binding capacity of the cationic AuNRs was evaluated by agarose gel electrophoresis. Even in the presence of a control pDNA (pCMV-DsRed), colloidal stability was only slightly decreased for all AuNRs, except the PLL-treated AuNRs (PLL-AuNRs) (Fig. 1c). Furthermore, the amount of free pCMV-DsRed gradually decreased with increasing amount of cationic AuNRs, except in the case of catHDL-treated AuNRs (catHDL-AuNRs) (Fig. 1e,f, Supplementary Fig. S3), clearly demonstrating that all AuNRs other than catHDL-AuNRs showed significant DNA binding capability. At an AuNR/DNA weight ratio of 10, more than 90% of the pCMV-DsRed added appeared to be bound to the cationic AuNRs, in all cases except the catHDL-AuNRs. Interestingly, the zeta potentials amongst the AuNR/pCMV-DsRed complexes at this ratio were dissimilar, despite using an equivalent amount of pCMV-DsRed (Fig. 1d). Only in the case of DOTAP-AuNRs, the zeta potential was significantly negatively shifted upon pCMV-DsRed binding. The reason for this apparent disparity is unclear at present, but these results may reflect possible differences in binding sites for pDNA, such as the outer surface or within the dispersant layer surrounding the AuNRs.

### pDNA transfection efficiency

**Figure 2:**
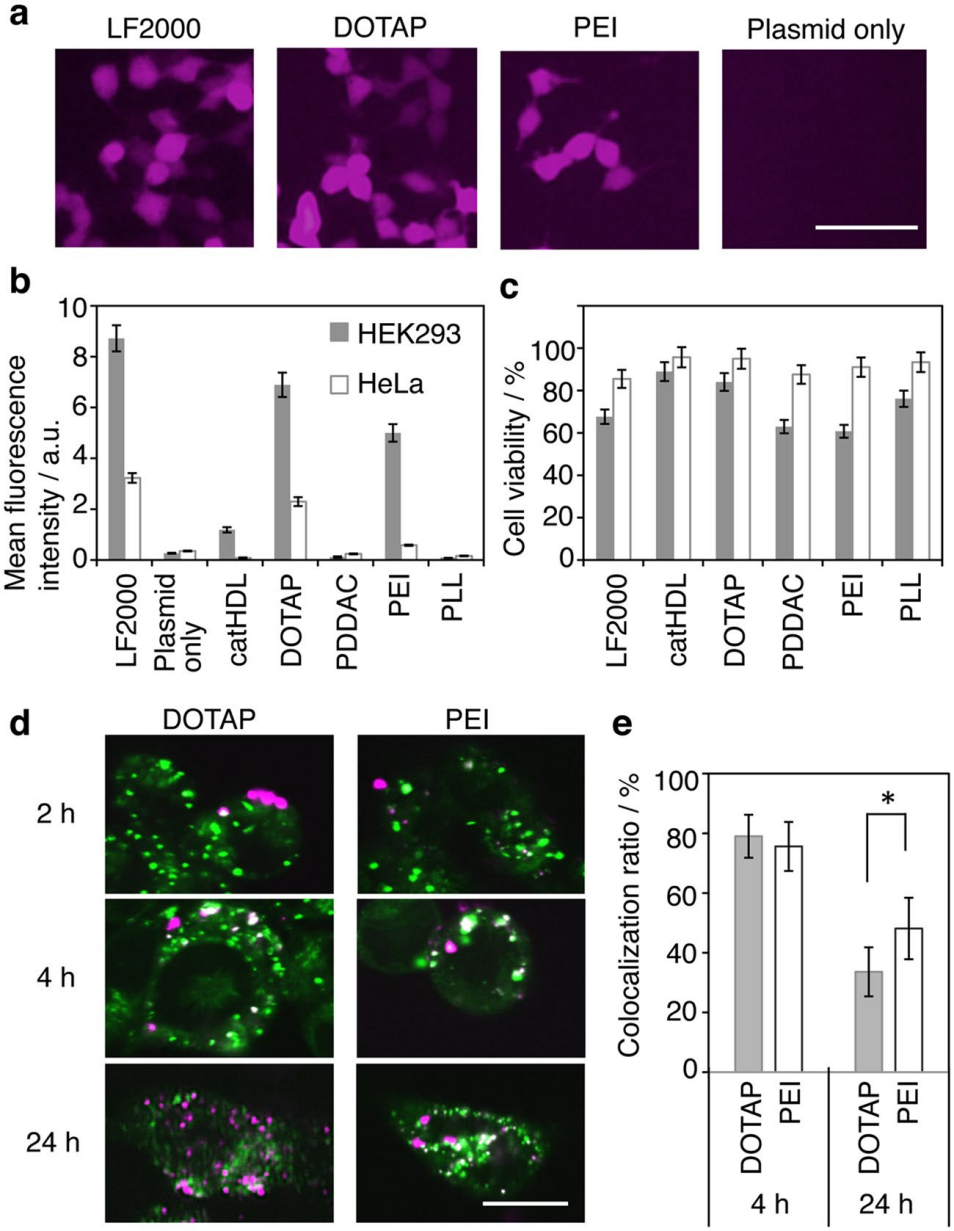
Intracellular gene delivery by cationic AuNRs. (**a**) Fluorescence images of HEK293T cells treated with cationic AuNR/pCMV-DsRed complexes. LF2000 was used as a positive control. Scale bar = 100 µm. (**b**) Transfection efficiency determined by flow cytometry analysis. Data indicate the mean fluorescence intensity of DsRed (n = 3, average ± SD). HEK293T cells and HeLa cells were treated with AuNR/pCMV-DsRed complexes ([Au] = 20 µg/mL, pCMV-DsRed = 2 µg/mL) for 24 h. DOTAP-AuNRs show a high transfection efficiency, comparable to that of LF2000. (**c**) Cell Count Kit-8 (CCK-8) assay data for HEK293T cells and HeLa cells treated with AuNR/pCMV-DsRed complexes for 24 h. Data indicate the mean cell viability (n = 3, average ± SD). The cytotoxicity of cells treated with DOTAP-AuNRs is lower than that of those treated with LF2000. (**d**) Time-dependent change of the localization of AuNRs in HEK293T cells. DOTAP- and PEI-AuNRs were labeled with Rho-PE and Alexa Fluor 546, respectively. AuNR localization (red signal) was analyzed after 2, 4 and 24 h treatment. Late endosomes/lysosomes were stained with LysoTracker Green DND-26 (LysoTracker, green). (**e**) The colocalization ratios of the red pixels to green pixels after 4 and 24 h were calculated, respectively (n = 10, average ± SD). Asterisks indicate P values < 0.05 determined using Student’s t-test. Scale bar = 10 µm.

The transfection efficiency of AuNR/pCMV-DsRed complexes was evaluated using HEK293T cells and HeLa cells. Lipofection by Lipofectamine 2000 (LF2000) was used as a positive control. After 24 h of treatment, only DOTAP-AuNRs and PEI-treated AuNRs (PEI-AuNRs) showed significant transfection abilities in both HEK293T cells and HeLa cells, as observed by DsRed expression (Fig. 2a,b). In particular, DOTAP-AuNRs showed a comparable efficiency to LF2000 (Fig. 2b), while showing a lower cytotoxicity than LF2000 (Fig. 2c). To the best of our knowledge, this is the first demonstration that AuNRs are comparable to LF2000 in terms of pDNA transfection efficiency.

To gain insight into the mechanism of the high transfection ability of DOTAP-AuNRs, DOTAP- and PEI-AuNRs were compared from various viewpoints. Under our transfection condition (20 µg/mL AuNRs), all pCMV-DsRed added was bound to the AuNR surface (Fig. 1e). When compared by inductively coupled plasma (ICP) analysis (Supplementary Fig. S4), cells were observed to take up 20% more DOTAP-AuNRs than PEI-AuNRs, indicating higher pCMV-DsRed delivery by DOTAP-AuNRs. Next, we investigated the intracellular localization of the two AuNRs by confocal microscopy. We observed that the red fluorescence signal, indicating AuNR localization, was localized at the plasma membrane after 2 h of transfection (Fig. 2d, Supplementary Fig. S5), and then moved from the plasma membrane to the late endosomes/lysosomes (green) by 4 h of transfection in both DOTAP- and PEI-AuNR-treated cells (Fig. 2d, Supplementary Fig. S5). The colocalization ratios of the red pixels to green pixels after 4 h were calculated to be 79.0 ± 7.2% for DOTAP-AuNRs and 75.6 ± 8.2% for PEI-AuNRs (Fig. 2e). After 24 h, the DOTAP- and PEI-AuNRs appeared to have exited from the late endosomes/lysosomes and were dispersed in the cytoplasm. The colocalization ratios of the red and green fluorescence signals at 24 h decreased to 33.6 ± 8.2 and 48.1 ± 10.3% for DOTAP- and PEI-AuNRs, respectively (Fig. 2e). These results indicate that the higher transfection efficiency of DOTAP-AuNRs may be, at least in part, due to the higher cell uptake and endosomal escape. While the mechanism of the higher cell uptake of the negatively charged DOTAP complexes compared to that of the positively charged PEI complexes (Fig. 1d) remains to be elucidated, the higher endosomal escape may be explained by the ability of oleate to function as a helper lipid^24^.

To investigate whether our surface modification method using oleate and DOTAP may be applicable for other nanoparticles, we similarly treated magnetite nanoparticles. When oleate-coated magnetite nanoparticles were mixed with DOTAP at a weight ratio of 20, a stable colloidal dispersion was obtained (Supplementary Fig. S6). The transition of the zeta potential from negative to positive suggested surface modification with DOTAP (Supplementary Fig. S6). Like DOTAP-AuNRs, DOTAP-treated magnetite nanoparticles also showed higher transfection efficiency than PEI-treated magnetite nanoparticles, and was comparable to that of LF2000 (Supplementary Fig. S6). These results clearly demonstrate that our method may be generally applicable for any oleate-coated nanoparticle.

Before the laser illumination experiments, we sought to optimize the conditions for DOTAP-AuNR preparation by changing the DOTAP/AuNR weight ratio, based on transfection efficiency. At mixing ratios lower than 10, the colloidal stability and transfection efficiency of DOTAP-AuNRs were reduced (Supplementary Fig. S7). At a higher mixing ratio of 20, DOTAP-AuNRs also showed lower transfection efficiency, despite higher colloidal stability. Thus, DOTAP-AuNRs prepared at a DOTAP/AuNR weight ratio of 10 were used for the following laser illumination experiments.

### Induction of protein expression by intracellular photothermal heating

**Figure 3:**
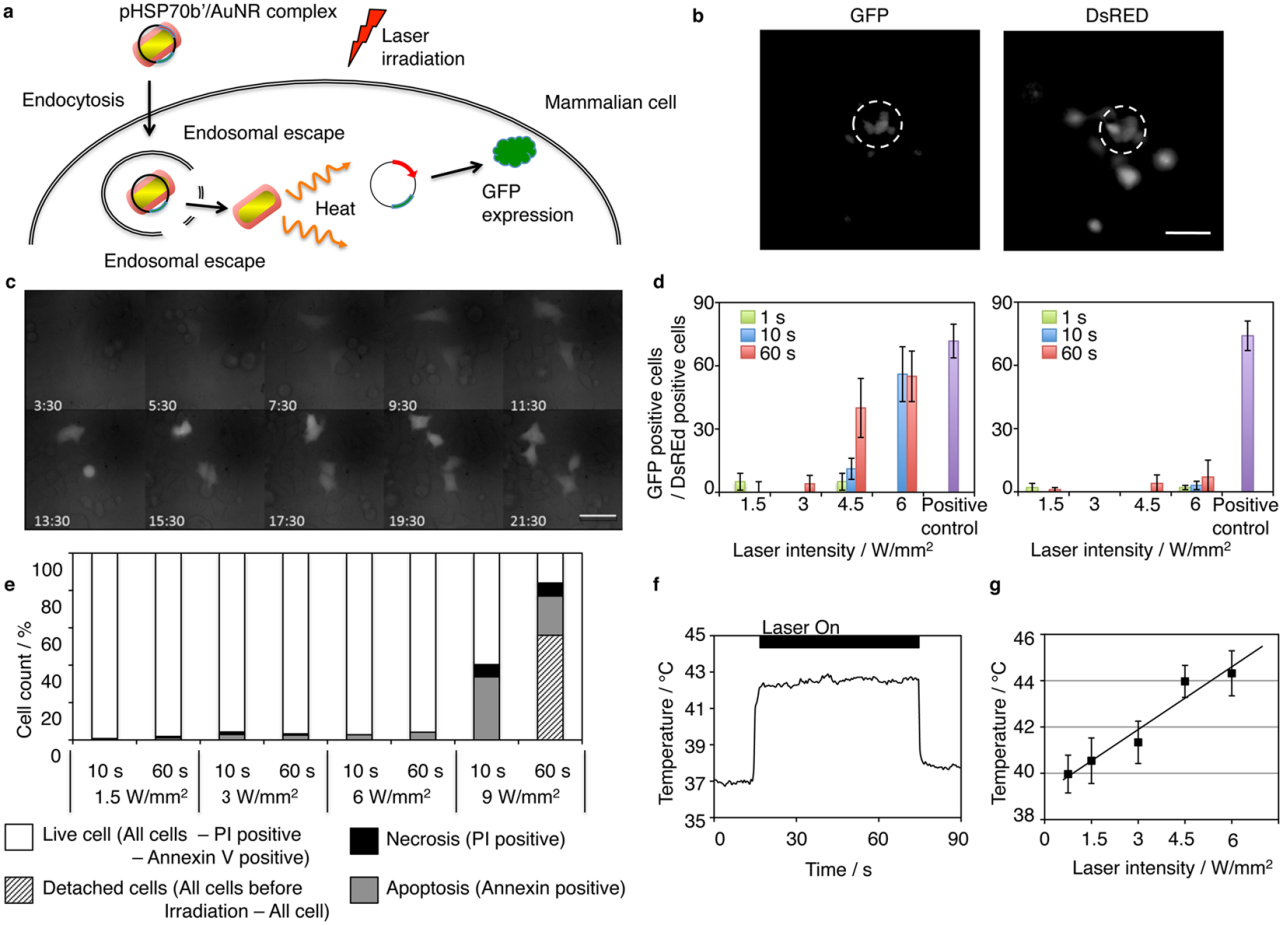
Intracellular photothermal heating of HEK293T cells by DOTAP-AuNRs. (**a**) Schematic of photothermal induction of protein expression by AuNRs. *HSP70b*’ promoter-driven expression vector (pHSP70b’) is transfected into cells by DOTAP-AuNRs and activated by brief NIR illumination. (**b**,**c**) Photoinduced EGFP expression in HEK293T cells. pHSP70-GFP and pCMV-DsRed were cotransfected by DOTAP-AuNRs. After 24 h, DsRed-positive cells were illuminated at 780 nm (6 W/mm^2^, 10 s). White broken circles indicate the illuminated area. Representative fluorescence images 24 h after illumination are shown in (**b**) and time lapse images after illumination are shown in (**c**). Scale bars = 100 µm in (**b**), 40 µm in (**c**). (**d**) Photoinduction efficiency for DOTAP-AuNRs (left) and LF2000 (right). Data were calculated based on the number of EGFP-positive cells/the number of DsRed positive (=photoinducible) cells within illuminated areas (n = 3, average ± SD). Data for the positive control were from cells incubated at 42 °C for 30 min (whole-cell heating). (**e**) Phototoxicity by intracellular photothermal heating. After illumination, cells were stained with Annexin-V and propidium iodine to detect apoptosis and necrosis, respectively. (**f**) Intracellular photothermal heating of AuNRs (780 nm, 6 W/mm^2^). The temperature around intracellular DOTAP-AuNRs was estimated utilizing the temperature dependence of Rho-PE fluorescence intensity (Fig. S9). Within a few seconds of illumination, the temperature reaches approximate 42 °C, which is the threshold temperature (42 °C) of *HSP70b*’ promoter activation. (**g**) Laser power intensity-dependence of the maximum temperature around illuminated AuNRs (n = 3, average ± SD).

We next performed photoinduced transgene expression using the DOTAP/AuNRs (Fig. 3a). We constructed a plasmid vector under the control of the human *HSP70b*’ promoter and inserted the EGFP gene (pHSP70-EGFP). HEK293T cells cotransfected with pHSP70-EGFP and pCAG-tdTomato using LF2000 exhibited only tdTomato expression under normal conditions, while they showed a significant increase in EGFP expression following heat shock at 42 °C for 30 min or longer (Supplementary Fig. S8). Next, pHSP70-EGFP and pCMV-DsRed were cotransfected in HEK293T cells using DOTAP-AuNRs. After 24 h, DsRed-positive cells in the targeted area (100 µm dia.) were illuminated at 780 nm using a NIR laser, and the resultant EGFP expression was evaluated after another 24 h. As shown in Fig. 3b, illuminated cells showed a marked increase in EGFP expression, whereas the surrounding off-target cells showed little to no EGFP expression. The induction of EGFP expression could be detected as soon as 6 h after NIR illumination by time-lapse imaging (Fig. 3c). When compared with the induction efficiency after heat-shock treatment at 42 °C for 30 min (positive control), maximal photoinduction was found to be achieved by illumination for 10 s at 6 W/mm^2^ (Fig. 3d, left panel). Under this laser condition, the temperature rise of the culture media was negligible and induction of EGFP expression was not detected in cells transfected using LF2000 (Fig. 3d, right panel). On the other hand, no significant changes were observed in the number of DsRed positive cells after illumination (Supplementary Fig. S9). Phototoxicity was low when cells were illuminated at 6 W/mm^2^, with less than 5% cells dying in 24 h. In contrast, cell death was significantly increased at 9 W/mm^2^ (Fig. 3e).

To estimate the intracellular temperature around the AuNRs during illumination, the temperature dependency of the fluorescence intensity of 1,2-Dioleoyl-sn-glycero-3-phosphoethanolamine-N-(lissamine rhodamine B sulfonyl) (Rho-PE) was utilized (Supplementary Fig. S10)^21^. Cells were treated with Rho-PE-labeled DOTAP-AuNRs and illuminated. The intracellular temperature quickly increased to reach a plateau of approximately 42 °C (Fig. 3f), which is the threshold temperature for activating the HSP70b’ promoter. When the maximum temperatures achieved at various laser power intensities were plotted, a linear relationship was observed (Fig. 3g). Based on this calibration curve, it was found that at laser power intensities of 4.5 W/mm^2^ or higher, the intracellular temperature could be increased to higher than 42 °C. These estimated temperature data agree well with the results in Fig. 3d, and demonstrate that intracellular photothermal heating by AuNRs is the main mechanism of HSP promoter-driven EGFP expression induction. These results clearly demonstrate that the HSP70b’ promoter can be safely driven by NIR illumination of the transfected carrier AuNRs in the cells.

**Figure 4:**
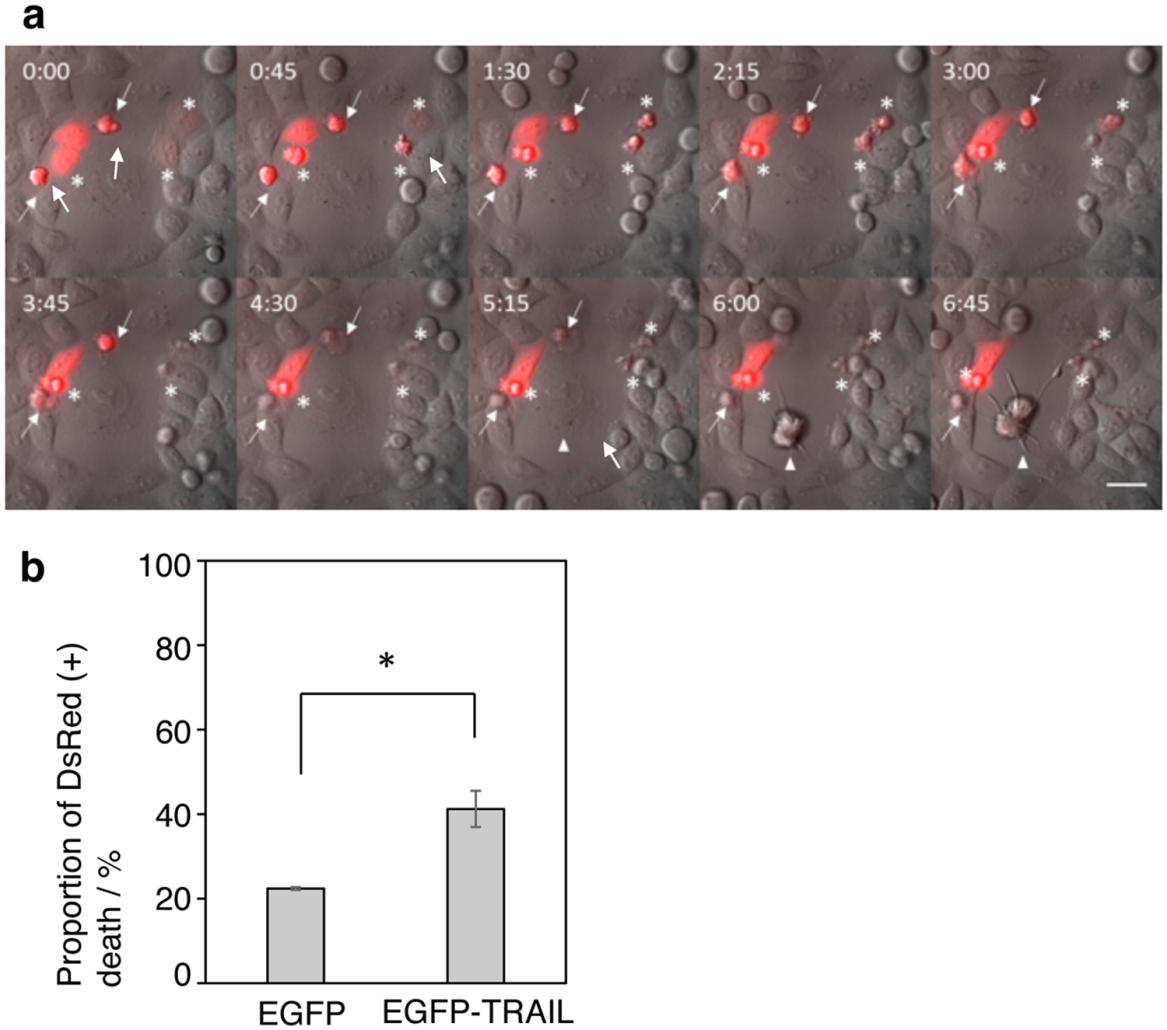
Light-induced elimination of cancer cells by DOTAP-AuNRs. HeLa cells were cotransfected with pCMV-DsRed and either pHSP70-EGFP or pHSP70-EGFP-TRAIL by DOTAP-AuNRs and photoinduced. (**a**) Montage shows time lapse of 45-minute interval of pHSP70-EGFP-TRAIL transfected cells (Supplementary Movie S1). Time 0:00 refers to start of time lapse. Time lapse started approximately 3 hours after laser irradiation. Arrows show DsRed positive cells that had died before start of time lapse imaging. Asterisks show DsRed positive cells that die during time lapse imaging. Arrowhead shows DsRed negative cell that dies during time lapse imaging. Scale bar = 40 µm. (**b**) Total number of DsRed positive cells and DsRed positive cell deaths were counted in time lapse imaging taken 3–20 hours post-photoinduction. GFP (n = 2), GFP-TRAIL (n = 3), 15–20 cells per experiment. *Indicates P < 0.05 with Student’s t-test.

As a proof of concept of the time- and site-specific activation of gene expression achievable by use of our system, we next attempted to develop a new method for therapeutic gene delivery to cancer cells. Tumor necrosis factor (TNF)-related apoptosis-inducing ligand (TRAIL) is a type-II transmembrane ligand that specifically induces apoptosis in many transformed cell lines by activating the death receptors DR4 and DR5, while having little effect on normal cells^25, 26^. We constructed an expression vector for an N’terminus-tagged eGFP-TRAIL fusion product, which has been shown to be effective in killing various tumor cell lines^27^, driven by the HSP70b’ promoter (pHSP70-EGFP-TRAIL) and transfected it into HeLa cells by LF2000. HeLa cells are derived from human cervical cancer and are thus sensitive to apoptotic activity of TRAIL^28^. We confirmed that heat shock induced significant cell death of HeLa cells transfected with pHSP70-EGFP-TRAIL, but not those with pHSP70-EGFP (Supplementary Fig. S11). Next, pHSP70-EGFP-TRAIL was cotransfected with pCMV-DsRed using DOTAP-AuNR and photoactivated by NIR laser under a time-lapse fluorescent microscope. DsRed-positive cells in the illuminated area (200 µm dia.) progressively showed typical morphological changes of apoptotic cells within 6 h of observation (Supplementary Movie S1). We also occasionally detected similar apoptotic changes of neighboring cells (arrowhead in Fig. 4a). The cell death rate of TRAIL-expressing cells was significantly higher than control cells (Fig. 4b), demonstrating that NIR-induced transgene expression, and not photoheating itself, could induce cell death. Thus, our DOTAP-AuNRs may have potential application in the molecular therapy of cancer

## Discussion

In the present study, we achieved photoinduction of transgene expression by brief illumination for 10 s, which is quite short considering that in the case of whole-cell heating, 30 min is necessary to induce comparable photoinduction (Supplementary Fig. S8). These results suggest that there may be some differences in the sensitivity of cells between intracellular localized heating and whole-cell heating. However, studies using similar NIR laser setups have reported that both brief (<1 s) and long (>10 min) illuminations were sufficient for activation of heat shock promoters^10–12^. Therefore, other experimental factors, e.g., photothermal heating conditions, may also have some effect on the duration of heating required for HSP promoter activation. One previous study utilized IR-LEGO as a source of illumination, which exploits direct heating of water molecules by 1480 nm laser illumination at >20,000 W/mm^2^. While IR-LEGO is a powerful technique available for activation of HSP promoters, to date, its application has been limited to transgenic cold-blooded animals or plants, which precludes simple comparison with our study using mammalian cells. Andersson *et al*. have demonstrated that PEI-coated AuNRs can induce plasmid-based HSP promoter-driven transgene expression in mammalian cells by 800 mW pulse-laser illumination of a comparable intensity (2 s at 5–15 W/mm^2^) to the present study (10 s at 6 W/mm^2^). On the other hand, two other studies utilized relatively long illuminations to activate HSP promoters; intracellular carbon nanohorns^10^, silica-gold nanoshells^11^, and hollow gold nanoparticles^11^ were illuminated with a continuous-wave (CW) laser for 30 min at 5 mW/mm^2^ or for 15 min at 20 mW/cm^2^, respectively. These experimental conditions are summarized in Table 1. Among the possible parameters that may affect the photothermal heating of the nanomaterials, laser emission mode may be excluded due to our observation of similar levels of *HSP70b*’ promoter activation by DOTAP-AuNRs illuminated by either pulsed or CW laser (Supplementary Fig. S12). Furthermore, considering that these nanomaterials have similar photothermal conversion efficiencies, it may be concluded that high laser power density is a key factor underlying the differences in reported required illumination time.

**Table 1:** Laser illumination parameters used in photoactivation studies with nanomaterials.

Laser parameter	DOTAP-AuNRs	PEI-AuNRs^7^	Carbon nanohorns^5^	NS^a,^ ^6^ and HGNP^b,^ ^6^
Mode	Pulse	Pulse	CW	CW
Intensity	6 W/mm^2^	5–15 W/mm^2^	5 mW/mm^2^	20 mW/mm^2^
Time	10 s	10 pulse (4 s)	30 min	15 min

^a^Silica-gold nanoshell, ^b^Hollow gold nanoparticle.

Illumination of cells at high laser power density increases the risk of phototoxicity (Fig. 3e). In the case of carbon nanohorns, illumination at a laser power density of higher than 5–12 mW/mm^2^, which is still much lower than the 6 W/mm^2^ in our study, significantly decreased cell viability. Thus, future work necessitates the clarification of why such high laser power densities were tolerated when using our DOTAP-AuNRs and Andersson’s PEI-coated AuNRs. We hypothesize that cytosolic localization of the photothermal nanomaterials is responsible for the safe photothermal heating at high power densities. DOTAP-AuNRs were mainly localized outside the late endosomes/lysosomes when illuminated (Fig. 2d), whereas carbon nanohorns were mainly inside them^10^.Andersson’s PEI-coated AuNRs may have also had a similar localization pattern to that of DOTAP-AuNRs, based on our results for PEI-AuNRs (Fig. 2d). Activation of the HSP promoter first requires activation of the HSF transcription factor, which under normal conditions is localized in the cytosol, via thermo-sensitive complex formation as well as release of inhibition by chaperon proteins^29^. Because the HSF activation machinery is located in the cytosol, nanoparticles sequestered in late endosomes/lysosomes may require balanced illumination allowing slow transfer of the generated heat to the cytosol, without causing fatal degeneration of the intracellular vesicles. Therefore, photothermal heating by DOTAP-AuNRs with intense, but brief, illumination may be more effective in activating the heat shock response, due to acute and localized cytosolic heating in the vicinity of HSF-activating machinery.

In conclusion, we have developed a new method for the surface modification of AuNRs with oleate and DOTAP, which enables simultaneous transfection of plasmid DNAs and heat-generating AuNRs at an unexpectedly high efficiency. The former function was also conferred on magnetite nanoparticles by this surface modification. This system using a new combination of three “old” components provides a unique opportunity for site-directed, light-inducible transgene expression in mammalian cells by a NIR laser, with minimal phototoxicity. By using this system, we have successfully induced cancer-cell specific apoptosis by NIR illumination-driven gene expression *in vitro*. It remains to be clarified to what degree of dependence heat shock pathway activation is influenced by the subcellular localization of heat generation. Further study is also needed on the delivery and photoactivation of DOTAP-AuNRs in tumor tissues *in vivo*. However, the precise spatiotemporal control of gene expression in mammalian cells by single transfection using DOTAP-AuNRs and NIR light will pave the way for applications in cell biology studies as well as less-invasive therapies.

## Methods

General reagents were purchased from Nacalai Tesque (Kyoto, Japan). Gold(III) chloride, sodium borohydride, PSS, PDDAC, PEI, and PLL were purchased from Sigma-Aldrich (Saint Louis, MO, USA). Silver nitride, L(+)-ascorbic acid, sodium oleate, 1-palmitoyl-2-oleoyl-*sn*-glycero-3-phosphocholine (POPC), sodium cholate, Gold ICP-MS Standard, and the lactate dehydrogenase (LDH) assay kit were obtained from Wako (Osaka, Japan). Rho-PE and DOTAP were purchased from Avanti Polar Lipids, Inc. (Alabaster, AL, USA). Opti-MEM, LF2000, LysoTracker Green DND-26 (LysoTracker), and fetal bovine serum (FBS) were obtained from Life Technologies (Carlsbad, CA, USA). *Escherichia coli* strain BL21 was purchased from Novagen (Madison, WI, USA). Spectra/Por Dialysis membranes (MWCO 50 kDa) were purchased from Spectrum Laboratories (Rancho Dominguez, CA, USA). NAP-5 columns were purchased from GE Healthcare UK Ltd. (Buckinghamshire, UK). Cell culture dishes and trypsin/EDTA were obtained from BD Biosciences (San Jose, CA, USA). Glass-based dishes with or without grid lines were purchased from Matsunami Glass Co. Ltd. (Osaka, Japan). Annexin V-FITC and propidium iodide were obtained from Funakoshi (Tokyo, Japan). Cell Counting Kit-8 was obtained from Dojindo (Kumamoto, Japan).

### Plasmids

pDsRed-monomer-N1 (pCMV-DsRed) was obtained from Takara Bio Inc. (Shiga, Japan). pCl-neo mammalian expression vector was obtained from Promega. (WI, USA). To generate the pHSP70-EGFP plasmid, a 450-bp DNA fragment containing the −456/−6 bp region (relative to translation start) of the human HSP70B’ gene *HSPA6* (NCBI Accession No. DQ521571) was amplified with AseI and HinDIII restriction sites from human genomic DNA by polymerase chain reaction (PCR) using primers #1 and #2 (see Table S1). The PCR product was digested and inserted into the AseI and HinDIII sites of pEGFP-N1 (catalog 6085-1, Clontech, CA, USA). pCAG-tdTomato, encoding tdTomato under control of the CMV enhancer and chicken β-actin promoter, was constructed as previously described^30^. Generation of the pHSP70-EGFP-TRAIL plasmid, whose expression results in a fusion protein of TRAIL tagged with EGFP at its N-terminus, was performed as follows. pHSP70-EGFP was digested with BamHI and NotI to remove the EGFP and stop codon. An EGFP fragment was amplified by PCR with primers #3 and #4 from pEGFP-N1. A TRAIL fragment was amplified by primers #5 and #6 from a human cDNA library (NCBI Accession No. U37518.1). The EGFP and TRAIL fragments were inserted in frame into the digested pEGFP-N1 vector using the In-Fusion HD cloning kit (Clontech, Mountain View, CA, USA). Correct construction of all plasmids was confirmed with sequence analysis.

### Preparation of AuNRs

AuNRs were prepared according to a seed-mediated growth method^22^. The seed solution was prepared by adding 600 µL of ice-cold 0.6 mM NaBH_4_ solution in 10 mL of HAuCl_4_ aqueous solution (HAuCl_4_, 0.01 mM; CTAB, 0.1 M). This solution was vigorously stirred at 30 °C for 30 min. The seed solution was then added to 30 mL growth solution. Synthesized AuNRs were centrifuged at 20,000 × *g* for 20 min and redispersed in 0.1 M CTAB solution to remove impurities.

### Preparation of cationized AuNRs

Cationic polyelectrolyte-coated AuNRs were prepared by a layer-by-layer assembly method^17^. As-prepared AuNR dispersion (0.1 mg/mL, 1 mL) was centrifuged at 20,000 × *g* for 20 min and redispersed in PSS solution (2 mg/mL PSS in 6 mM NaCl, 10 mL). After stirring at room temperature for 3 h, the dispersion was centrifuged at 20,000 × *g* for 10 min and redispersed in deionized H_2_O (1 mL). PSS-treated AuNRs (PSS-AuNRs) were centrifuged at 20,000 × *g* for 10 min and redispersed in each polyelectrolyte solution (2 mg/mL in 6 mM NaCl, 10 mL). Each AuNR dispersion was stirred at room temperature for 3 h to allow coating of PSS-AuNRs with the cationic polyelectrolytes, and then washed with PBS to remove free polyelectrolytes. catHDL and catHDL-AuNRs were prepared as described elsewhere^21, 23^. Briefly, a cationic peptide (YGRKKRRQRRR)-fused apoA-I truncate, without its first 43 amino acids of the N-terminal, and a mixture of POPC and DOTAP at a molecular ratio of 7:3 were mixed at a lipid/protein molar ratio of 250. The resulting catHDL was purified by dialysis and centrifugation. As-prepared AuNRs were treated with sodium oleate in deionized H_2_O. Oleate-AuNRs were incubated at 50 °C for 1 h with catHDL at a protein/Au weight ratio of 0.4:1 to yield catHDL-AuNRs. The catHDL-AuNRs were then washed with PBS to remove free catHDL.

DOTAP-AuNRs were prepared by simply mixing oleate-AuNRs and 10 mg/mL DOTAP, which was solubilized in PBS containing 30 mg/mL sodium cholate at 37 °C for 2 h, at a weight ratio of 1:10. The mixture was dialyzed against PBS to remove sodium cholate. DOTAP-AuNRs were washed with PBS to remove any debris.

### Characterization of AuNRs

UV/vis/NIR absorption spectra were measured with a JASCO V-730 spectrometer (Tokyo, Japan). The zeta potential of all AuNRs was measured in 10 mM Tris-HCl (pH 7.0) with a Malvern Zetasizer Nano Z (Worcestershire, UK).

### Cell culture

Human embryonic kidney HEK293T cells and HeLa cells were maintained in DMEM supplemented with 10% FBS, 100 U/mL penicillin, and 100 µg/mL streptomycin. Cells were cultured in 5% CO_2_ and 95% humidified air at 37 °C and passaged every 2–3 days.

### Plasmid DNA (pDNA) transfection by cationic AuNRs

Cells were seeded in 96-well plates at 2 × 10^4^ cells/well and incubated for 24 h before transfection. AuNR/pDNA complexes were prepared in 100 µL Opti-MEM by simple mixing of 1 µg pCMV-DsRed and 10 µg of AuNRs, and incubation at room temperature for 20 min. Lipofection using LF2000 was performed according to the manufacturer’s protocol. LF2000 (1 µL) and pCMV-DsRed (1 µg) were mixed in 25 µL Opti-MEM at room temperature for 20 min. The mixture was diluted with cell culture media (75 µL) and added to each well without purification. After incubation for 24 h, cells were harvested with trypsin/EDTA, centrifuged, and resuspended in culture media. Transfection efficiency for resuspended cells was evaluated by a Millipore Guava easyCyte flow cytometer (MA, USA). Cell viability was evaluated using a Cell Count Kit-8 and a Molecular Devices Spectra Max M2 microplate reader (Sunnyvale, CA, USA).

### Intracellular localization

Intracellular localization of AuNR/pDNA complexes was evaluated by a Fluoview FV10i confocal microscope (Olympus, Tokyo, Japan). Fluorescently labeled DOTAP-AuNRs were prepared by mixing oleate-treated AuNRs and 10 mg/mL DOTAP containing 1 mol% Rho-PE, which was solubilized in PBS containing 30 mg/mL sodium cholate at 37 °C for 2 h, at a weight ratio of 1:10. Fluorescently labeled PEI-AuNRs were prepared by mixing PEI-AuNRs and an amine-reactive fluorescent dye, Alexa Fluor 546 NHS ester (Thermo Fisher, Waltham, MA, USA), in 0.1 M sodium bicarbonate at a w/w ratio of 100:1. Free Alexa Fluor 546 NHS Ester was removed by NAP-5 columns. AuNR/pDNA complex were prepared by using cationized AuNRs and pCl-neo vector plasmid. HEK293 cells were seeded in glass-based dishes at 2 × 10^4^ cells/cm^2^ and treated with fluorescently labeled DOTAP-AuNR/pDNA or PEI-AuNR/pDNA complexes in the same manner as the pDNA transfection experiments described above. After 2, 4, or 24 h, the late endosomes/lysosomes were stained with LysoTracker.

### Activation of gene expression by heat and light

AuNR/pDNA complexes were prepared in 100 µL Opti-MEM by mixing 1 µg of pHSP70-EGFP), 0.5 µg of pCMV-DsRed and 10 µg of AuNR solution. Cells were seeded in glass-based dishes with an imprinted grid (Iwaki, Shizuoka, Japan) at 2 × 10^4^ cells/cm^2^. Cells were transfected with pDNAs or AuNR/pDNA complexes as described above and incubated for 24 h at 37 °C. Heat shock was carried out by replacing the culture media with prewarmed (42 °C) media and incubating the dishes in a CO2 incubator at 42 °C for 10–60 min, as indicated. For NIR illumination, cells were washed with fresh media and a circular area approximately 200 µm in diameter was illuminated at 780 nm for 10–60 s at 50–300 mW (1.5~9 W/mm^2^) using an ECLIPSE Ti epifluorescence microscope (Nikon, Tokyo, Japan) equipped with 20× objective lens (NA 0.75, Nikon), a beam expander, a Chameleon femtosecond-pulsed laser (Coherent, CA, USA), and an ORCA-flash 4.0 digital camera (HAMAMATSU, Shizuoka, Japan). Cells were fixed after 24 h incubation for evaluation of DsRed and EGFP expression in the illuminated area by an FV1000 fluorescence microscope (Olympus). For time-lapse imaging, cells were placed in an LCV100 incubator microscope (Olympus) with a 20× objective lens (NA 0.7, Olympus) immediately following illumination, and imaged at 10–15 min intervals for 18 h.

### Phototoxicity assay

The cells were fixed at 24 h after NIR laser illumination and stained with annexin V-FITC and propidium iodine according to the manufacturer’s protocol. The percentages of live, early apoptotic, and late apoptotic/necrotic cells were determined by counting over 100 cells in the illuminated area.

### ICP analysis of cationic AuNRs in cells

The cells were incubated for 24 h after NIR laser illumination and then scraped after washing with PBS twice. Harvested cells were incubated in aqua regia for 10 min, and dried on a block heater. The residue was dissolved in 1000 µl of 1 M HCl and Au content was quantified by a Shimadzu plasma atomic emission spectrometer ICPE-9000 (Kyoto, Japan).

### Intracellular temperature measurement

DOTAP-AuNRs were fluorescently labeled with 1 w/w% Rho-PE and transfected into HEK293T cells by the protocol described above. Under illumination at 780 nm, the temperature rise around the AuNRs inside the cell was estimated by measuring the fluorescence intensity of Rho-PE using a fluorescent microscope (Eclipse Ti, Nikon). Fluorescence intensity data were converted to temperature data using a linear calibration curve obtained for a Rho-PE labeled AuNR dispersion in PBS.

## Electronic supplementary material

Supplementary information

Supplementary Movie S1

## References

[1] Cambridge SB, Davis RL, Minden JS (1997). Drosophila mitotic domain boundaries as cell fate boundaries. Science.

[2] Cambridge SB (2009). Doxycycline-dependent photoactivated gene expression in eukaryotic systems. Nat. Methods.

[3] Wang X, Chen XJ, Yang Y (2012). Spatiotemporal control of gene expression by a light-switchable transgene system. Nat. Methods.

[4] Polstein LR, Gersbach CA (2015). A light-inducible CRISPR-Cas9 system for control of endogenous gene activation. Nat. Chem. Biol..

[5] Halfon MS, Kose H, Chiba A, Keshishian H (1997). Targeted gene expression without a tissue-specific promoter: Creating mosaic embryos using laser-induced single-cell heat shock. Proc. Natl. Acad. Sci. USA..

[6] Halloran MC (2000). Laser-induced gene expression in specific cells of transgenic zebrafish. Development.

[7] Ramos, D. M., Kamal, F., Wimmer, E. A., Cartwright, A. N. & Monteiro, A. Temporal and spatial control of transgene expression using laser induction of the hsp70 promoter. *BMC Dev*. *Biol*. **6** (2006). 10.1186/1471-213X-6-55 PMC1664555 17116248

[8] Kamei Y (2009). Infrared laser-mediated gene induction in targeted single cells *in vivo*. Nat. Methods.

[9] Miyako E (2012). Photothermic regulation of gene expression triggered by laser-induced carbon nanohorns. Proc. Natl. Acad. Sci. USA..

[10] Cebrian V (2013). Enhancing of plasmonic photothermal therapy through heat-inducible transgene activity. Nanomedicine.

[11] Andersson HA, Kim YS, O’Neill BE, Shi ZZ, Serda RE (2014). HSP70 promoter-driven activation of gene expression for immunotherapy using gold nanorods and near infrared light. Vaccines (Basel).

[12] Lyu Y, Xie C, Chechetka SA, Miyako E, Pu K (2016). Semiconducting polymer nanobioconjugates for targeted photothermal activation of neurons. J. Am. Chem. Soc..

[13] Richter K, Haslbeck M, Buchner J (2010). The heat shock response: life on the verge of death. Mol. Cell.

[14] Weissleder R (2001). A clearer vision for *in vivo* imaging. Nat. Biotechnol..

[15] Chen CC (2006). DNA-gold nanorod conjugates for remote control of localized gene expression by near infrared irradiation. J. Am. Chem. Soc..

[16] Huang HC, Barua S, Kay DB, Rege K (2009). Simultaneous enhancement of photothermal stability and gene delivery efficacy of gold nanorods using polyelectrolytes. ACS Nano.

[17] Xu L (2012). Surface-engineered gold nanorods: promising DNA vaccine adjuvant for HIV-1 treatment. Nano Lett..

[18] Wang FH (2014). Efficient, dual-stimuli responsive cytosolic gene delivery using a RGD modified disulfide-linked polyethylenimine functionalized gold nanorod. J. Control. Release.

[19] Hildebrandt B (2002). The cellular and molecular basis of hyperthermia. Crit. Rev. Oncol. Hematol..

[20] Brade AN (2000). Heat-directed gene targeting of adenoviral vectors to tumor cells. Cancer Gene Ther..

[21] Nakatsuji H (2015). Thermosensitive ion channel activation in single neuronal cells by using surface-engineered plasmonic nanoparticles. Angew. Chem. Int. Ed..

[22] Nikoobakht B, El-Sayed MA (2003). Preparation and growth mechanism of gold nanorods (NRs) using seed-mediated growth method. Chem. Mater..

[23] Murakami T (2014). Mesoscopic metal nanoparticles doubly functionalized with natural and engineered lipidic dispersants for therapeutics. ACS Nano.

[24] Wang XM (2013). Enhanced hepatic delivery of siRNA and microRNA using oleic acid based lipid nanoparticle formulations. J. Control. Release.

[25] Walczak H (1997). TRAIL-R2: A novel apoptosis-mediating receptor for TRAIL. EMBO J..

[26] Pan GH (1997). The receptor for the cytotoxic ligand TRAIL. Science.

[27] Kagawa S (2001). Antitumor Activity and Bystander Effects of the Tumor Necrosis factor-related apoptosis-inducing ligand (TRAIL) Gene. Cancer Res..

[28] Lalaoui N (2011). TRAIL-R4 promotes tumor growth and resistance to apoptosis in cervical carcinoma HeLa cells through AKT. PLoS One.

[29] Westerheide SD, Raynes R, Powell C, Xue B, Uversky VN (2012). HSF transcription factor family, heat shock response, and protein intrinsic disorder. Curr. Protein Peptide Sci..

[30] Fujishima K, Horie R, Mochizuki A, Kengaku M (2012). Principles of branch dynamics governing shape characteristics of cerebellar Purkinje cell dendrites. Development.

