# Supplementary material for: Surface chemistry for cytosolic gene delivery and photothermal transgene expression by gold nanorods
Source: Sci Rep. 2017 Jul 5;7:4694. doi: 10.1038/s41598-017-04912-1 (PMC5498644; doi:10.1038/s41598-017-04912-1)
Supplement: Supplementary file 1 — Supplementary information [file 41598_2017_4912_MOESM1_ESM.pdf]

Supplementary information

Surface chemistry for cytosolic gene delivery and photothermal transgene expression by gold nanorods

Author: Hirotaka Nakatsuji[†#], Kelly K. Galbraith[‡#], Junko Kurisu[§], Hiroshi Imahori[†,§], Tatsuya Murakami*[,§,¶] and Mineko Kengaku*[,‡,§]

Author affiliation

[†]Department of Molecular Engineering, Graduate School of Engineering, Kyoto University, Nishikyo-ku, Kyoto 615-8510, Japan

[‡]Graduate School of Biostudies, Kyoto University, Sakyo-ku, Kyoto 606-8501, Japan

[§]Institute for Integrated Cell-Material Sciences (WPI-iCeMS), Kyoto University, Sakyo-ku, Kyoto 606-8501, Japan

[¶]Department of Biotechnology, Faculty of Engineering, Toyama Prefectural University, Kurokawa 5180, Imizu City, Toyama 939-0398, Japan

# These authors equally contribute to this study

*E-mail address: kengaku@icems.kyoto-u.ac.jp, murakami@pu-toyama.ac.jp

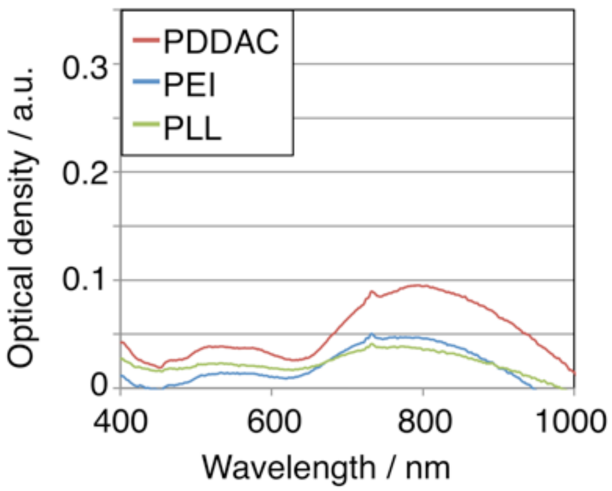

Figure S1. UV-*vis*-NIR absorption spectra of oleate-AuNRs treated with cationic polyelectrolyte in PBS ([Au] = 20 µg/mL). Compared with DOTAP-AuNRs (Fig. 1b), all AuNRs show broadened plasmon peaks in the NIR region, suggesting their aggregation.

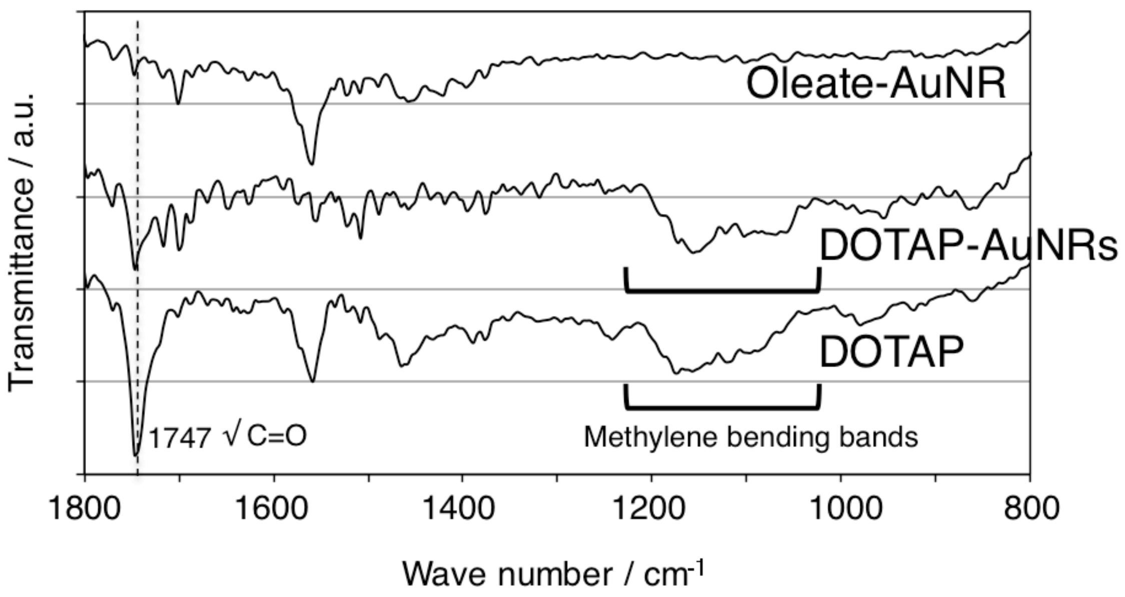

Figure S2. FT-IR spectra of DOTAP-AuNRs and two surfactants used for preparation. The signals derived from DOTAP can be seen in the DOTAP-AuNR spectra.

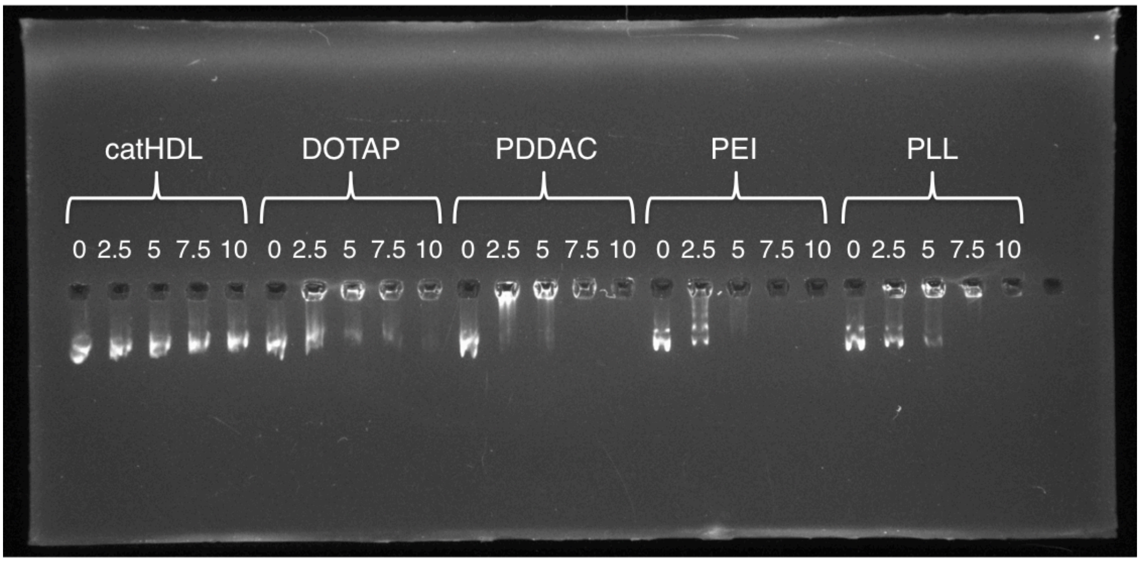

Figure S3. Full length agarose gel image corresponding to Fig. 1e. Numbers in the image indicate the w/w ratios of AuNRs to pCMV-DsRed.

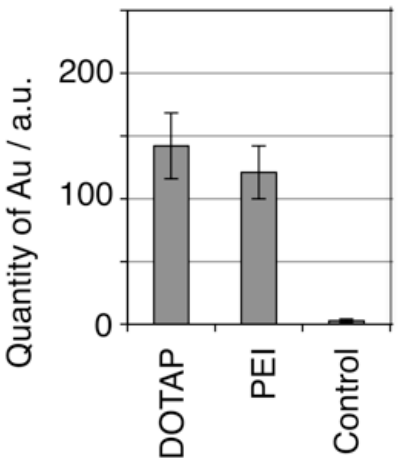

Figure S4. ICP analysis of AuNRs taken up by cells. HEK293T cells were treated with either DOTAP-AuNR/pCMV-DsRed or PEI-AuNR/pCMV-DsRed ([Au] = 20 μg/mL, pCMV-DsRed = 1 μg/mL) for 24 h. After treatment, cells were washed with PBS twice, scraped and incubated in aqua regia for 1 h to dissolve the AuNRs. The reaction solution was evaporated and dissolved in 1 M HCl. Au ions in solution were then quantified (n=3, average ± SD).

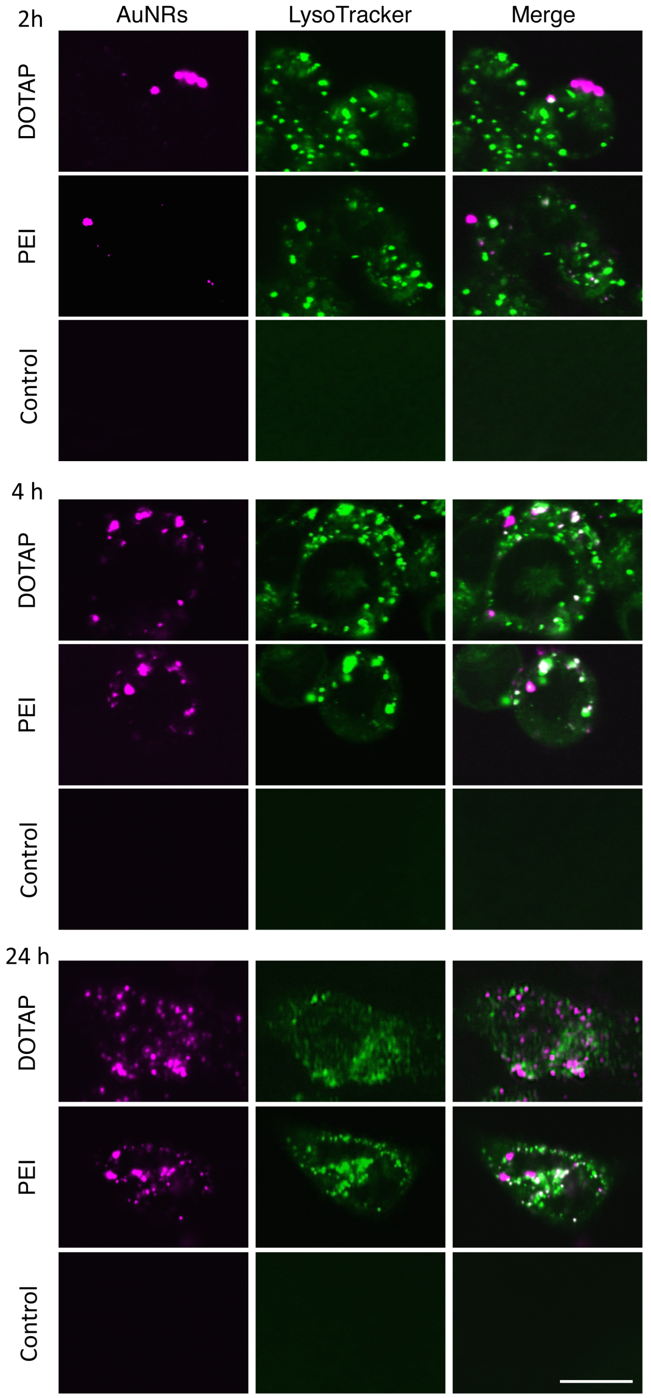

Figure S5. Fluorescence images corresponding to Fig. 2d. Scale bar = 10 μm.

a

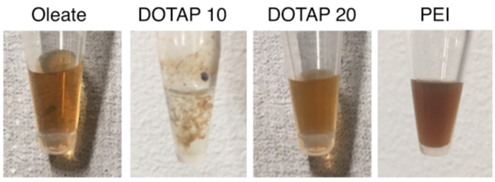

b

	Size / nm
Oleate	71 ± 5
DOTAP20	173 ± 14
PEI	225 ± 35

c

	Zeta potential/ mV
Oleate	- 51.3 ± 2.1
DOTAP10 (DOTAP/mg = 10)	35.4 ± 1.9
DOTAP20 (DOTAP/mg = 20)	28.6 ± 6.2
PEI	35.8 ± 1.3

d

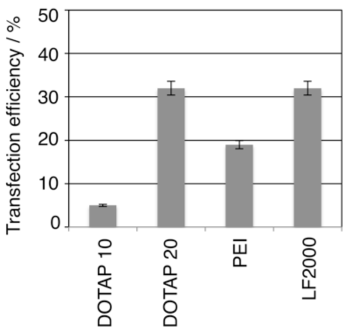

Figure S6. Applicability of our method to magnetite nanoparticles (a) Photographs of the dispersion of magnetite nanoparticles treated with oleate followed by DOTAP or PEI in PBS. Only dispersants used for the final treatment are indicated on the photographs. “DOTAP 10” and “DOTAP 20” indicate magnetite nanoparticles treated with DOTAP at 10 and 20 w/w ratios of DOTAP to magnetite nanoparticles, respectively. (b) Size and (c) zeta potential data (n=3, average ± SD) of magnetite nanoparticles. The size analysis of DOTAP 10 was not included due to massive precipitation, as shown above. (d) Transfection efficiency of magnetite nanoparticles by flow cytometry analysis (n=3, average ± SD). HEK293T cells were treated with magnetite nanoparticles/pCMV-DsRed complex ([Magnetite] = 20 μg/mL, pCMV-DsRed = 2 μg/mL) for 24 h.

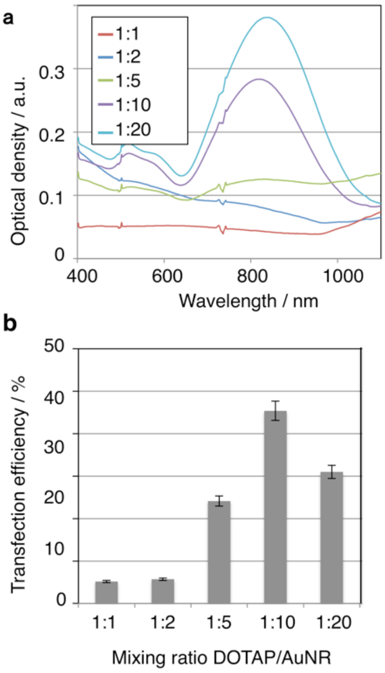

Figure S7. Optimization of DOTAP-AuNR preparation. (A) UV-*vis*-NIR absorption spectra of DOTAP-AuNRs prepared at various w/w ratios of DOTAP to oleate-AuNRs in PBS ([Au] = 20 µg/mL). Numbers in the inset indicate the ratios. At a ratio of 10 or more, DOTAP-AuNRs are stabilized. (B) Transfection efficiency of DOTAP-AuNRs determined by flow cytometry analysis. HEK293T cells were treated with DOTAP-AuNR/pCMV-DsRed complexes ([Au] = 20 µg/mL, pCMV-DsRed = 2 µg/mL). Data indicate the mean fluorescence intensity of DsRed (n=3, average ± SD). DOTAP-AuNRs prepared at a ratio of 10 show the highest transfection efficiency.

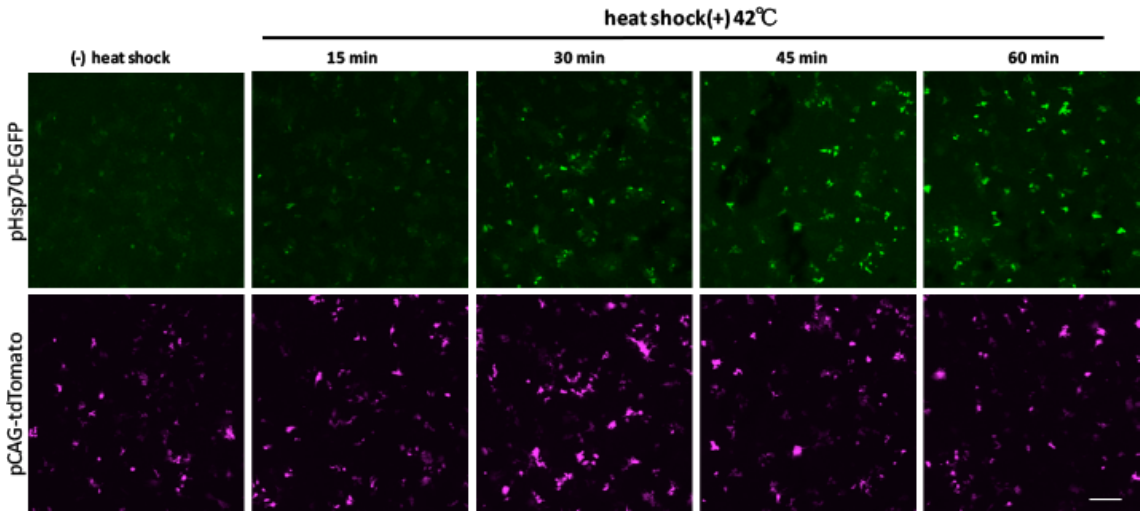

Figure S8. Influence of heating time on HSP promoter-driven protein expression. HEK293T cells cotransfected with pHSP70-EGFP and pCAG-tdTomato using LF2000. The cells were incubated at 42°C for the indicated times. After further incubation at 37°C for 24 h, cells that had been heated for 30 min or longer show significant EGFP expression.

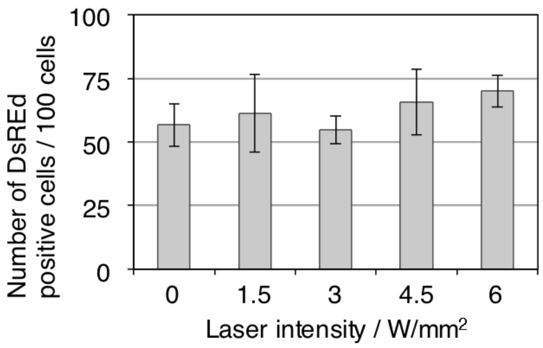

Figure S9. Effect of laser illumination on the number of DsRed positive cells after transfection with DOTAP-AuNRs in Fig. 3d (n=3, average ± SD)

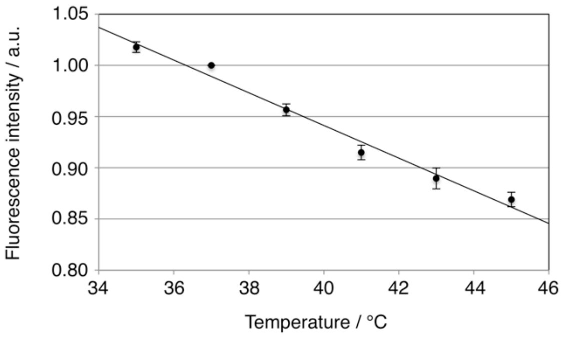

Figure S10. Calibration curve for local temperature measurement based on fluorescence intensity. Temperature dependency of Rho-PE fluorescence intensity for DOTAP-AuNR in PBS (pH 7.4) is shown. Data were obtained during heating and cooling processes between 35-45°C (n=3, average ± SD). Relative fluorescence intensities were calculated by dividing each peak value by the peak value at 37°C. The slope was calculated to be 1.60 %/°C.

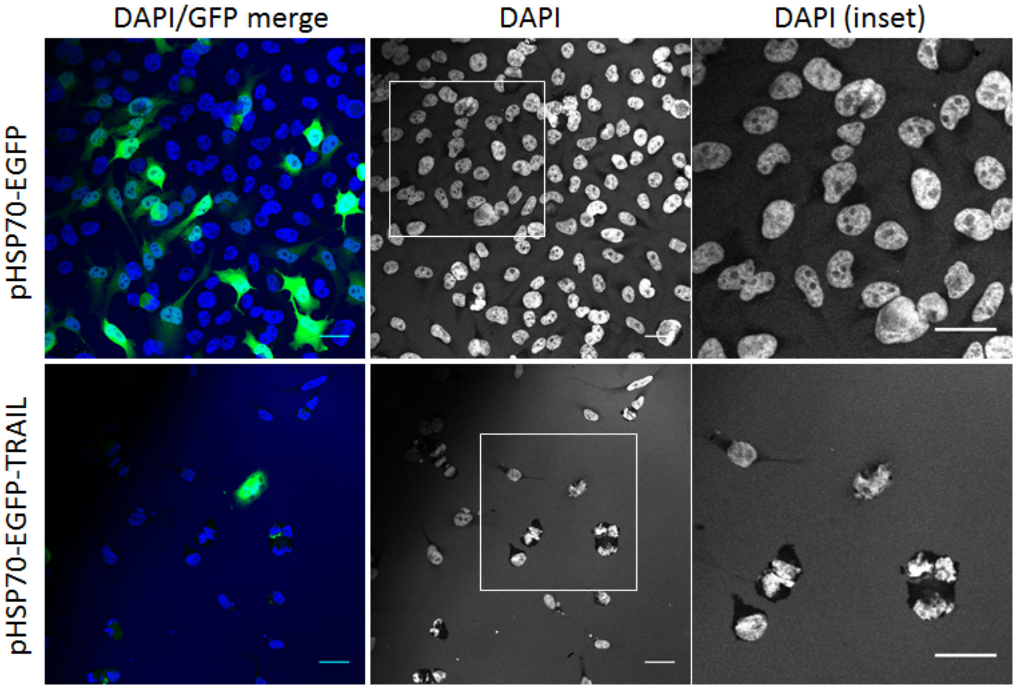

Figure S11. Observation of HeLa cell death driven by TRAIL expression. HeLa cells transfected with either pHSP70-GFP or pHSP70-GFP-TRAIL by Lipofectamine 2000 were heat shocked for one hour at 42ºC. Six hours later, cells were washed, fixed (4% PFA), and permeabilized. Cells were stained with primary antibody chick anti-GFP (1:1000) and secondary antibody Alexa Fluor 488, and post stained with DAPI 0.1 ug/mL. Images show that in the pHSP70-GFP-TRAIL condition, most of the cells had died and were washed off. The remaining cells show DAPI staining indicative of dead or dying cells.

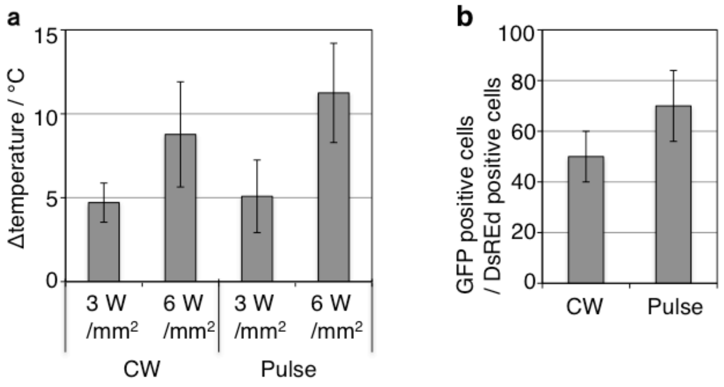

Figure S12. Effect of mode of 780-nm laser (fs-pulse vs CW) on intracellular photothermal heating. (a) Temperature achieved around intracellular AuNRs (n=5, average ± SD). Temperature data were estimated in the same manner as in Figure 4e. (b) Photoinduced EGFP expression in HEK293T cells (n=3, average ± SD). Cells were treated with DOTAP-AuNR/pHSP70-EGFP/pCMV-DsRed in the same manner as in Figure 4 (780 nm, 6 W/mm^2, 10 s). Differences between fs-pulse and CW in both (a) and (b) are not statistically significant.

Table S1 Primers used in this study.

No.	Name	Sequence
1	forward AseI 5' Hsp70b'	CGAattaatCCTCTAAAGTTGCTGCTTTTGCAGCC
2	reverse HinDIII 3' Hsp70b'	tataAAGCTTCTTGTCGGATGCTGGAGGCCACGGA
3	forward 5' pHSP70b' BamHI eGFP	gtaccgcgggcccgggatccaccggtcgccaccat
4	reverse 3' eGFP + 5' TRAIL	ctccatcatagccatCTTGTACAGCTCGTCCATGC
5	forward 3' eGFP + 5' TRAIL	TGGACGAGCTGTACAAGatggctatgatggaggtc
6	reverse pHSP70b' 3' TRAIL	tctagagtcgcggccgcTTAgccaactaaaaagg

Movie S1. Time lapse movie of HeLa cells cotransfected with pHSP70-EGFP-TRAIL and pCMV-DsRed using DOTAP-AuNR, and photoactivated by NIR laser. Time 0:00 refers to start of time lapse. Time lapse started approximately 3 hours after laser irradiation. Arrows show DsRed positive cells that had died before start of time lapse imaging. Asterisks show DsRed positive cells that die during time lapse imaging. Arrowhead shows DsRed negative cell that dies during time lapse imaging. Scale bar =40 µm. Images were acquired every 15 min for 7 hr. Playback rate is 8 fps.
